# Triple A Syndrome–A Rare Hereditary Cause of Achalasia

**DOI:** 10.14309/crj.0000000000001686

**Published:** 2025-04-25

**Authors:** Shivangini Duggal, Monica Botros, Emerald Zaw, Swati Mahapatra, Jesus Guzman, Keith Garrison, Richard Mccallum

**Affiliations:** 1 Department of Internal Medicine, Texas Tech University Health Sciences Center, El Paso, TX; 2 Texas Tech University Health Sciences Center Paul L. Foster School of Medicine, El Paso, TX; 3 Department of Internal Medicine, Division of Gastroenterology, Texas Tech University Health Sciences Center, El Paso, TX

**Keywords:** Allgrove syndrome, triple A syndrome, achalasia, alacrimia, adrenal insufficiency, dysphagia

## Abstract

Triple A syndrome (TAS) is a rare disorder inherited in an autosomal recessive manner. It is characterized by the triad of alacrimia, achalasia, and adrenal insufficiency. It is caused by mutations in the *AAAS *gene (achalasia-addisonianism-alacrima syndrome), which disrupts the protein ALADIN (Alacrima-Achalasia-Adrenal insufficiency Neurologic disorder protein), which plays an important role in nucleocytoplasmic transport and cellular stress response. Unlike the presented cases, most patients with TAS present in early childhood with various symptoms including dry eyes, difficulty swallowing, and recurrent infections in addition to signs of adrenal crisis such as hypotension, hypoglycemia, and hyperpigmentation. Diagnosis can be achieved by genetic testing, revealing the mutations in the *AAAS *gene. Management of TAS mainly focuses on addressing each condition separately such as prescribing artificial tears for alacrimia, glucocorticoid replacement therapy for adrenal insufficiency, and performing pneumatic dilation or surgical intervention for achalasia. Early diagnosis is crucial for improving quality of life and minimizing the morbidity and mortality linked to the syndrome.

## INTRODUCTION

Triple A syndrome (TAS), also known as Allgrove syndrome, is a rare autosomal recessive disorder that was initially identified in 1978.^1^ The disease is characterized by a triad of achalasia, alacrimia, and adrenal insufficiency. It can also be associated with autonomic dysfunction; hence, the term 4A syndrome has been suggested.^2^ Most cases have *AAAS *genemutation (achalasia-addisonianism-alacrima syndrome) located on chromosome 12q13.^3^ Establishing the diagnosis of TAS is difficult given the variable phenotypes and mutations.

TAS is a multisystem disorder characterized by 3 cardinal symptoms: achalasia, adrenal insufficiency, and alacrimia. In addition, neurological abnormalities including motor neuron-like disease or autonomic dysfunction might be present. It is an autosomal recessive disease caused by a mutation in the *AAAS *gene on chromosome 12q13, which encodes ALADIN protein (alacrima-achalasia-adrenal insufficiency neurologic disorder protein)^4^ This protein is involved in various functions such as cytoskeleton assembly and control of cell division. This mutation was identified in more than 90% of patients with TAS.^4^ Diagnosis can be confirmed using molecular testing; however, it is rarely used when there is a high clinical suspicion such as the presence of 2 out of the 3 cardinal symptoms.^5^ In the presented case series, we report 2 adult-onset cases of TAS.

## CASE REPORT

### Case 1

A 65-year-old man with a medical history of dry eyes, and chronic adrenal insufficiency on fludrocortisone and hydrocortisone since 2015, initially presented in 2019 with dysphagia to both solids and liquids for 3 years. Esophagogastroduodenoscopy (EGD) revealed food in the middle and lower thirds of the esophagus. Manometry showed aperistalsis and a nonrelaxing lower esophageal sphincter (LES), establishing the diagnosis of type II achalasia. The patient underwent a pneumatic dilation using a 30-mm size balloon and a gastrografin swallow postprocedure showed no evidence of perforation and an open LES. The patient reported complete resolution of his dysphagia and acid reflux. In 2023, the patient presented with recurrent dysphagia, muscle weakness, and dry eyes for a 6-month duration. Laboratory tests showed normal complete blood count, comprehensive metabolic panel, erythrocyte sedimentation rate, C-reactive protein, in addition to antinucleated antibody, and anti-Sjogren antibodies were negative. The patient underwent repeat EGD with dilation. The patient did not undergo genetic testing.

### Case 2

A 26-year-old woman with medical history of dry eyes and chronic adrenal insufficiency on dexamethasone 2 mg, since 2020, initially presented in 2016 with dysphagia, for which she had an EGD with evidence of monilial esophagitis. In addition, she underwent an esophageal motility study, which showed aperistalsis with hypertonic LES, establishing the diagnosis of type I achalasia. The patient underwent balloon dilation with a 30-mm and then a 35-mm diameter balloon 1 year later. The patient had recurrent of symptoms in 2019 and 2020 and underwent EGD twice with 100 units of botulinum toxin injected into the LES and lower third of the esophagus on each occasion. In 2023, the patient had an Eso-Functional Lumen Imaging Probe procedure with dilatation of LES for ongoing dysphagia. The patient did not undergo genetic testing.

## DISCUSSION

TAS is typically described in children, but there are reported cases of adult-onset TAS in the literature manifesting mainly with neurological symptoms.^6,7^ Alacrimia usually presents in early infancy, which is noted by the lack of tears when crying.^8^ Schirmer test is used to formally diagnose reduced or absent tear production.^9^ Artificial tears or lubricants are used to alleviate symptoms as well as prevent keratopathy and corneal ulceration.^10^

Achalasia is characterized by failure of relaxation of the LES accompanied by a loss of peristalsis in the smooth muscle of the body of the esophagus. It manifests later in life and is rare during childhood.^11,12^ Symptoms of dysphagia and regurgitation present similarly between patients with idiopathic achalasia and those with TAS; however, those with TAS have a higher LES pressure and association with more treatment failure.^13^ Achalasia diagnosis is made with esophageal manometry as the gold standard. Barium swallow can be suggestive but nonspecific and may even be interpreted as normal in up to one thirds of patients.^14^ EGD is essential to rule out other causes of dysphagia and can be used for treatment modalities like pneumatic balloon dilation, botulinum injection, and per-oral endoscopic myotomy if symptoms persist.^15^

Achalasia is present in approximately 57%–83% of patients with TAS at the time of diagnosis.^16^ In a study by Bakr et al, they describe TAS in 9 pediatric patients and the manometry findings showed impaired LES relaxation and disrupted esophageal motility. LES pressure was variably affected, with some patients exhibiting hypertonic LES, whereas others had normal LES pressure. Notably, esophageal peristalsis was severely impaired, with a lack of normal propagation in most cases and abnormal peristaltic wave amplitude in most patients. In adults, idiopathic achalasia is defined by esophageal aperistalsis and impaired LES relaxation in response to swallowing. However, in children, although absent LES relaxation is a universal feature, complete esophageal aperistalsis is uncommon.^17^ In comparison to previously published data on TAS, our patients demonstrated similar but distinct esophageal motility abnormalities on manometry, barium swallow, and EGD. Both patients exhibited impaired LES relaxation and reduced esophageal motility, consistent with achalasia. However, there were notable differences in their esophageal distensibility indices, dilation responses, and esophageal clearance on imaging. In the first case with type II achalasia, initial functional lumen imaging probe (FLIP) distensibility indices were markedly low (2.59 mm^2^/mm Hg at 40 mL, improving to 4.12 mm^2^/mm Hg postdilation). While in the second case with type I achalasia, FLIP testing before dilation showed slightly better initial distensibility (4.6 mm^2^/mm Hg at 20 mL, increasing to 6.5 mm^2^/mm Hg postintervention). The variability in distensibility indices and contraction patterns in our patients suggests a spectrum of disease severity, further supporting the idea that achalasia in TAS exhibits a distinct pathophysiology, possibly influenced by underlying autonomic dysfunction and progressive neurodegeneration. Newer high-resolution manometry studies may provide further insights into the unique esophageal motor patterns seen in 3A compared to IA.^18^

The underlying mechanism of achalasia in TAS appears to involve a combination of neurodegenerative, fibrotic, and possibly autoimmune processes affecting the enteric nervous system. Histopathological studies of the cardia in TAS reveals fibrotic enlargement of the intermuscular plane, a striking reduction in myenteric ganglia, and a severe loss of neuronal nitric oxide synthase expression, which is crucial for LES relaxation. The absence or marked reduction of nitrergic inhibitory signaling likely results in sustained LES hypertonicity, contributing to impaired esophageal emptying. In addition, the presence of CD3^+^ T-lymphocyte infiltrates in the myenteric plexus in some patients suggests a possible immune-mediated mechanism, further supporting the hypothesis that inflammatory or autoimmune factors may contribute to the progressive degeneration of enteric neurons. Interestingly, although interstitial cells of Cajal, which play a role in neuromuscular transmission, appear largely preserved in most TAS cases, their occasional reduction in some patients suggests that secondary alterations in the enteric pacemaker system may also contribute to dysmotility. The selective involvement of the esophageal myenteric plexus in TAS, in contrast to the normal histopathologic findings in the pylorus, implies that the pathophysiology of achalasia in TAS is distinct from primary idiopathic achalasia, potentially reflecting a disease-specific neurodevelopmental or degenerative process linked to mutations in the *AAAS *gene encoding the ALADIN protein.^19^

Adrenal insufficiency is seen in 85% of patients and is usually present in the first decade of life, and it is secondary to resistance to adrenocorticotropic hormone.^20^ It was thought initially that TAS is a variant of familial glucocorticoid deficiency, which was recognized later to be a separate entity.^21^ Undiagnosed adrenal insufficiency is considered a major cause of death due to severe hypoglycemia.^11,17^ Diagnosis is made by corticotropin stimulation test and measuring fasting morning cortisol and adrenocorticotropic hormone levels.^4,5^ Adrenal dysfunction is not limited to glucocorticoid deficiency as there are reported cases of TAS with aldosterone deficiency, which was independent of the extent of the glucocorticoid deficiency.^4,22^ Management of adrenal insufficiency includes replacement therapy, which is commonly in the form of hydrocortisone and additional fludrocortisone if there is associated mineralocorticoid deficiency.^4^ It is recommended to have lifelong follow-up with an endocrinologist although there are no formal guidelines for surveillance for those with TAS syndrome.^5^

Associated neurological symptoms typically manifest later in life following the other symptoms.^22^ Neurologic features in TAS include dysfunction of the corticospinal tracts, peripheral neuropathy, or motor neuron dysfunction.^23^ There have been reports of patients with TAS with amyotrophic lateral sclerosis-like symptoms including upper and lower motor neuron involvement.^24^ Other neurological abnormalities reported are autonomic dysfunction and ophthalmic symptoms such as optic atrophy and abnormal pupillary reflex.^25,26^ Nerve conduction studies from affected patients have shown sensorimotor polyneuropathy with axonal degeneration and demyelination.^27^

The treatment of TAS-related achalasia differs from idiopathic achalasia due to underlying fibrosis, neuronal nitric oxide synthase deficiency, and poorer response to dilation. In idiopathic achalasia, standard treatments include pneumatic dilation (PD), peroral endoscopic myotomy (POEM), and Heller myotomy with fundoplication.^28^ PD is effective in Type II IA (idiopathic achalasia), but its long-term success declines to 40%–50%, with repeated sessions increasing the risk of fibrosis and perforation.^29^ POEM offers precise myotomy and long-term symptom relief but carries a high risk of gastroesophageal reflux disease, managed with proton pump inhibitors. POEM has advantages over PD, as it allows for the creation of a submucosal tunnel, providing an endoluminal workspace where endoscopists can precisely adjust the length and depth of the incision based on the degree of obstruction at the cardia and lower esophagus. In addition, this technique facilitates easy biopsy collection from the muscular layer, offering valuable histopathological insights for further treatment.^30^

In contrast, TAS-related achalasia is more refractory to PD due to severe fibrosis, limiting its long-term efficacy. Although some patients with TAS improve after a single PD session, evidence supporting sustained benefit is lacking. POEM is the preferred approach in TAS, offering customized myotomy and biopsy collection for further evaluation. Advanced POEM techniques, including T-incision and Hook knife use, have improved efficiency and reduced complications.^31^ Studies on achalasia treatment indicate that 83% of patients undergoing POEM experience long-term symptom relief without requiring reintervention. However, mild symptom recurrence may begin in the second year, and 17% of patients eventually require additional procedures due to worsening symptoms over time.^32–34^ In TAS-related achalasia, where long-term treatment outcomes remain poorly studied, regular follow-up is essential to monitor disease progression and identify cases requiring repeat intervention. Gastroesophageal reflux disease remains a concern, with reflux esophagitis occurring in some patients with TAS post-POEM, requiring acid suppression therapy.^35^ Given the rarity of TAS, long-term follow-up and further studies are needed to refine treatment strategies and assess POEM's durability in this population.

In conclusion, TAS is a rare disorder characterized by achalasia, alacrimia, and adrenal insufficiency, with achalasia being a major symptom that significantly impacts patients' quality of life. Although alacrimia typically presents in infancy, achalasia and adrenal insufficiency develop later, making early diagnosis challenging. Achalasia in TAS is often associated with higher LES pressures and poor response to PD, necessitating endoscopic or surgical myotomy. Given its profound effect on swallowing function and daily life, recognizing TAS is crucial for gastroenterologists to ensure timely diagnosis and management. Achalasia should raise suspicion for TAS, especially when accompanied by other features discussed in this case series. Although a definitive cure remains elusive, refining POEM techniques and optimizing treatment strategies are essential to improving long-term outcomes and enhancing the quality of life for patients with TAS.

## DISCLOSURES

Author contributions: M. Botros, E. Zaw, S. Mahapatra, S. Duggal: wrote the manuscript and conducted the literature review. J. Guzman, K. Garrison and R. McCallum: critically reviewed and approved of the article. All authors reviewed and approved of the whole manuscript. R. Mccallum is the article guarantor.

Financial disclosure: None to report.

Informed consent was obtained for this case report.
